# Sustainable Utilization of Mushroom By-Products Processed with a Combined Osmotic Dehydration Pretreatment and a Hot-Air-Drying Step

**DOI:** 10.3390/foods13091339

**Published:** 2024-04-26

**Authors:** Natalia A. Stavropoulou, Andriana E. Lazou, Maria C. Giannakourou

**Affiliations:** 1 Laboratory of Chemistry, Analysis & Design of Food Processes, Department of Food Science and Technology, School of Food Sciences, University of West Attica, Agiou Spyridonos St., Egaleo, 12243 Athens, Greece; nstavropoulou@uniwa.gr (N.A.S.); alazou@uniwa.gr (A.E.L.); 2 School of Chemical Engineering, Laboratory of Food Chemistry and Technology, National Technical University of Athens, Zografou Campus, 9, Iroon Polytechniou Str., Zografou, 15772 Athens, Greece

**Keywords:** hurdle technology, mushroom by-products, dehydration, response surface methodology, valorization, drying kinetics

## Abstract

Mushroom production and consumption are gaining increased interest due to their unique flavor and nutritional value. However, in the mushroom industry, large amounts of by-products are generated, which have a high negative environmental and economic impact. In this study, an osmotic dehydration process followed by hot-air-drying was applied to mushroom stems to produce dried mushrooms as the end product. The osmotic dehydration conditions (concentration of hypertonic solution, specifically, 10–30% maltodextrin and 20–40% oligofructose; a treatment time of 40–80 min; and a temperature range of 30–50 °C) were optimized using response surface methodology (RSM). The results showed that a four-factor three-level Box–Behnken experimental design was effectively implemented to evaluate the effect of the process parameters and identify the optimal osmotic dehydration conditions for producing osmotically dehydrated mushrooms. The main factor affecting mass transfer was the osmosis temperature, and the optimal conditions were found to be 38 °C, 40% oligofructose and 19.3% maltodextrin as the osmotic agents, and 80 min of immersion time. Moreover, the results showed that osmotic pretreatment, in the optimal conditions, significantly reduced the required drying time of the by-products compared to traditional hot-air-drying, especially at milder drying temperatures. Consequently, the required energy was also reduced by at least 40% at 50 °C.

## 1. Introduction

In recent years, industrial ecology concepts such as the circular economy, have been considered best practices for innovation. They involve recycling waste to create new food products and applications. In this context, it is interesting to consider that, in the mushroom industry, a significant amount of by-products is generated and rejected (approximately 60 million tons of mushroom waste are produced worldwide each year, which accounts for 5–20% of the total production); this has a negative impact on the environment and increases industry expenses [1,2]. These by-products could be in the form of caps or stipes but can also include mushrooms that are not of an acceptable caliber, shape, or size or spent mushroom substrate (SMS). These by-products are valuable in many applications and possess an equally high nutritional value to that of commercial mushrooms [1,3,4,5]. A large percentage of mushroom by-products (mostly SMS) are used in animal feed formulations due to improved economic viability, ecological considerations, and animal quality. Other potential applications of mushroom by-products are for food uses, as fertilizers and partial substrates for mushroom growth, for biological treatments, for food supplementation, in the cosmetics and cosmeceuticals industry, etc. [3,5].

In this work, the potential use of mushroom stems rejected from *Pleurotus ostreatus* cultivation has been studied. Practically, the mushroom stem is the underground growing part of the mushroom, which serves as a route for nutrient absorption from the growth medium. The total waste after commercial treatment accounts for about 30% of the total fruiting body mass [1], and, of all the cultivated mushrooms, Pleurotus accounts for around 19% of the global production [3,5]. The utilization of mushroom stems to produce new functional foods is, so far, an underexplored field.

Osmotic dehydration (OD) is a mild technique widely applied as a pretreatment in the fruit and vegetable industry. This process includes the immersion of a food in hypertonic solutions, and, therefore, the simultaneous incorporation of solutes and the removal of water are obtained. Some parameters that affect the OD process are the osmotic agent, the time and temperature of osmosis, and the physicochemical characteristics of the food sample [6,7]. OD has been applied as a pretreatment before the air-drying of pumpkins [8], pineapples [9], carrots [10], beetroots [11], broccoli [12], and mushrooms [13,14] to produce functional snacks with a high quality and a high nutritional value. Katsoufi et al. (2021) [8] used sweeteners (sucrose and oligofructose) to osmodehydrate pumpkins before a final step of air-drying at temperatures of 40, 60, and 80 °C. Sensory tests revealed a preference for the products osmotically dehydrated with oligofructose, thus producing low-calorie candied fruit. Maleki et al. (2020) [10] studied the effect of osmosis on carrot, followed by air-drying, combining an impregnation step with polyphenolic compounds using process optimization. The optimal conditions were found to be 60 min of osmosis with a solution of 60% sucrose and a drying temperature of 70 °C. Biswas et al. (2022) [9] investigated the combination of different osmotic pretreatments (1% trehalose, 2% NaCl, 10% fructose, and 10% sucrose) and drying temperatures to reduce sensory modifications on dried pineapples. The drying temperatures tested were 50, 55, and 60 °C. The results showed that a 1% trehalose solution for 30 min and air-drying at 55 °C were the optimal conditions. Md Salim et al., in 2019 [12], used the OD as a pretreatment, followed by microwave-assisted hot-air-drying, in order to produce dried broccoli stalks. They found that the osmotically dehydrated product had better color retention and a softer texture compared to a fresh food sample. 

Regarding studies on mushrooms, Ashtiani et al., in 2023 [13], applied a preliminary cold plasma treatment and then immersed fresh white button mushrooms in 30% glucose and 1% calcium hydroxide solutions (*w/w*), at 40 °C for 120 min, aiming to increase mass transfer phenomena. The samples were then placed inside a convective dryer at an air temperature of 50 °C and a velocity of 1.0 m/s. The results showed that using cold plasma and OD prior to convective drying significantly reduced the time of the drying step, contributing to lower energy consumption and a better product quality. González-Pérez et al., in 2019 [14], studied the effect of osmotic dehydration (25% NaCl, 80 °C, and a 180 min duration of osmosis) prior to convective drying (50, 65, and 80 °C drying temperature, velocity 2.0 m/s) on the mass transfer, shrinkage, and shape characteristics of white mushroom pilei. According to the results, the predicted water diffusivities were significantly lower with the OD pretreatment, compared to those when fresh samples were used. Kurozawa et al., in 2012 [15], mostly focused on the alternative modeling of the drying kinetics of fresh and osmotically pretreated mushroom slices (*Agaricus blazei*), with OD being performed at ambient conditions, into a 10% salt solution. Similarly, Singla et al., in 2010 [16], applied OD in a 5% salt solution, followed by the addition of spices and vacuum-drying, aiming at producing a spicy snack food. The specific goal of this work was to study the antioxidant activities and polyphenolic properties of the end products. Nonetheless, an exhaustive literature research revealed that there are only a few studies that applied OD as a pretreatment followed by hot-air-drying on mushrooms, during which the osmotic agent used was either a conventional carbohydrate or salt; in our study, aiming to produce a final dehydrated product of acceptable sensory attributes and increased functional properties, inulin, a well-known prebiotic dietary fiber, was used in the osmotic solution. Oligofructose is a type of inulin, which is a subgroup of fructans. It consists of polymers with a degree of polymerization (DP) of 10 or less, leading to a rather small molecular weight. Oligofructose is highly soluble and can be used to increase the dietary fiber content of foods without affecting their organoleptic characteristics. It has a lower caloric value than standard carbohydrates due to the β (2-1) bonds linking the fructose molecules, making it a suitable option for diabetics [17,18]. On the other hand, maltodextrin of a low molecular weight (DE in the range between 10 and 21) is a carbohydrate commonly used as a food additive and an osmotic agent frequently used for lowering water activity, mainly for fruit and vegetables being osmotically treated [6,19,20,21]. As also demonstrated in Shinde & Ramaswamy (2022, 2023) [22,23], the incorporation of maltodextrins of low DE (as the one employed in this study) is favorable to limit solid uptake and maximize moisture reduction. Taking into account that maltodextrin is a carbohydrate of a high glycemic index, meaning that it can raise the blood sugar levels quickly after consumption, it is important to control and limit its concentration in the final product. In fact, some researchers suggest that consuming small amounts of maltodextrin (such as the ones found in our osmodried end product) may have beneficial effects for people with type 2 diabetes, such as gradually improving postprandial hyperglycemia [21,24]. Besides testing the effect of different osmotic agents, another aim of our work was the optimization of the OD process, based on response surface methodology (RSM) principles, in order to assess the optimized conditions before applying the drying step.

Another aspect of our research was the sustainable exploitation of mushroom production side streams; mushroom by-products (mostly including stems and pieces of a non-uniform shape) have been mostly studied as sources of bioactive compounds [2,25,26,27]; however, to our knowledge, no investigation has been conducted to date on the production of new functional foods from mushrooms by-products. So, another aim of this work was the valorization of *Pleurotus osteatus* mushroom stems to develop a novel dehydrated product with superior-quality characteristics. Finding ways to utilize mushroom by-products is vital for environmental conservation, economic development, and the promotion of sustainable practices in the food industry. It aligns with the broader goal of reducing waste and promoting the efficient use of resources, contributing to a more sustainable and responsible approach to food production and consumption. The novelty of this work lies in the valorization of *Pleurotus osteatus* by-products as potential raw materials for the development of a novel snack. The production procedure consisted of an osmotic dehydration pretreatment and a subsequent air-drying step. The osmotic dehydration process parameters (process temperature, time, and solution concentration) were analyzed and optimized using the principles of response surface methodology (RSM). The optimally produced OD mushroom stems were subsequently air-dried at different temperatures (50, 60, and 70 °C), and the mass transfer kinetics were mathematically described using a first-order kinetic model. The total drying time to produce the final novel mushroom products was also estimated.

## 2. Materials and Methods

### 2.1. Sample Preparation

**Figure 1:**
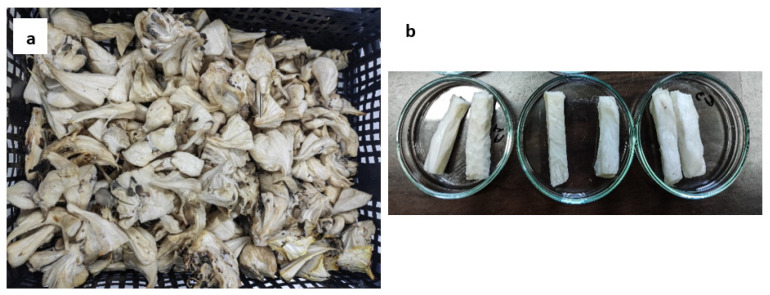
(**a**) Raw mushroom by-products and (**b**) mushroom by-products after cutting.

Fresh oyster mushroom stems (Figure 1), considered as by-products and rejected for animal feed, were obtained from the local company “DIRFIS MUSHROOMS” with an initial humidity of 88.72 ± 1.62% (wet basis). The moisture content was measured using the vacuum-oven method at 70 °C (Heraeus Instruments Vacutherm, ThermoScientific, Waltham, MA, USA) for 24 h, according to the official method of AOAC (1990) [28]. The mushroom stems were washed to remove dirt, dried on a blotting paper, and cut into rectangular pieces of a uniform size and shape (~5 × 0.5 × 0.5 cm, ~6 ± 1 g). The samples were then immersed in freshly prepared osmotic solutions, as described in detail in the following section.

### 2.2. Osmotic Dehydration Process

Food-grade maltodextrin (Maltodekstrin, DE 12–20, A.D. industrija skroba “JABUKA”, Pančevo, Serbia), oligofructose (chicory oligofructose, COSUCRA Groupe Warcoing), and food-grade ascorbic acid (ascorbic acid, a.g., Penta) were purchased from local providers. The process parameters under investigation, which affect the OD product quality, were osmotic solution type, process temperature, and time. The selected OD solutions were combinations of maltodextrin at ratios of 10, 20, and 30% and oligofructose at ratios of 20, 30, and 40%, as shown in Table 1. The proportions were selected according to preliminary experiments and previously published results [29]. The OD temperature levels selected were 30, 40, and 50 °C, while the immersion time ranged up to 120 min. Moreover, all the osmotic solutions contained 5% NaCl and 1.5% ascorbic acid to prevent enzymatic browning reactions [30]. After preliminary experiments, the ratio of the sample to the osmotic solution was set to 1:15 (*w*/*w*) to avoid any dilution of the osmotic solution, which would reduce the driving force for mass transfer phenomena. The OD process was performed in a water bath (PolyScience water bath, WB10A11B) for constant temperature control. After the OD procedure, the OD mushroom stems were rinsed with water to remove the excess solution and wiped carefully with absorbent paper. All the experiments were performed in triplicate.

### 2.3. Mass Transfer Calculations

Water loss (WL) and solid gain (SG) were calculated according to the following equations (Equations (1) and (2)):

(1) $$WL=\frac{{(M}_{0}-m_{0})-(M-m)}{m_{0}}$$

(2) $$SG=\frac{\left(m-m_{0}\right)}{m_{0}}$$

where *M*_0_ is the initial mass of fresh material before the OD, *M* is the mass of the mushroom samples after time t of OD, *m* is the dry mass of the mushrooms after time t of OD, and *m*_0_ is the dry mass of the untreated material [31,32].

### 2.4. Physicochemical Properties’ Determination during Osmotic Treatment

The water activity of the mushroom by-products and the °Brix of the hypertonic osmotic solutions were determined by an a_w_-meter (AquaLab Dew Point Water Activity Meter 4TE, METERGroup, Inc., Pullman, WA, USA) and a hand-held refractometer (Atago, Master refractometer, Fukayai, Japan), respectively. The color of the samples was measured with a tristimulus chromatometer (Handy Colour Tester, Model H-CT, SUGA Test Instruments, Tokyo, Japan). The CIELAB color scales were used, with coordinates (L*, a*, b*) being directly read from the chromameter. The total color change ΔE* and chroma (C*) were calculated according to Equations (3) and (4), respectively:

(3) $$\Delta {Ε}^{*}=\sqrt{{\left(L_{t}^{*}-L_{0}^{*}\right)}^{2}+{\left(a_{t}^{*}-a_{0}^{*}\right)}^{2}+{\left(b_{t}^{*}-b_{0}^{*}\right)}^{2}}$$

(4) $$C^{*}=\sqrt{{\left(a_{t}^{*}-a_{0}^{*}\right)}^{2}+{\left(b_{t}^{*}-b_{0}^{*}\right)}^{2}}$$

where L*, a*, and b* are the luminosity, redness, and yellowness of the samples, respectively. Subscripts “t” and “0” refer to time t and zero time, respectively [31,32,33]. All the measurements were performed in triplicate.

A texture analyzer (TA-XT2i of Stable Micro Systems, Godalming, UK) was used for the texture analysis of all the samples, and a TPA (texture profile analysis) test was carried out. The test was performed according to the method described in Stavropoulou et al., in 2022 [34]. Briefly, a 6 mm cylindrical compression probe and a 25 kg load cell were used. The experiment was run under the subsequent instrument specifications: pretest speed—5 mm/s; test speed—2 mm/s; and post-test speed—5 mm/s at a 50% deformation. Texture characteristics such as hardness (F_max_) were calculated according to the initial value of the untreated samples (F_max0_). Hardness is defined as the maximum force of the first compression on the TPA curve. Each measurement was carried out in three replicates to estimate the average value and standard deviation.

### 2.5. OD Experimental Design

Response surface methodology (RSM) was used to investigate the effect of hypertonic solution concentration (10–30% maltodextrin and 20–40% oligofructose), osmosis temperature (30–50 °C), and immersion time (40–80 min) to obtain the optimal osmotic process parameters for mushroom by-products. The choice of carbohydrates was based on previous comparative research on alternative osmotic agents [35,36,37].

RSM is a statistical tool used for multiple regression analysis using quantitative data taken from specially designed experiments. A Box–Behnken design with four factors at three levels including twenty-seven experiments (DOE response surface application, Minitab^®^ 17.1.0, Philadelphia, PA, USA) formed by three central points was used (Table 1), and the indices measured included the mass transfer parameters and physicochemical and selected quality indices. The following polynomial model was used for the data:

(5) $$Y=b_{0}+b_{1}X_{1}+b_{2}X_{2}+b_{3}X_{3}+b_{4}X_{4}+b_{11}X_{1}^{2}+b_{22}X_{2}^{2}+b_{33}X_{3}^{2}+b_{44}X_{4}^{2}+b_{12}X_{1}X_{2}+b_{13}X_{1}X_{3}+b_{14}X_{1}X_{4}\;+b_{23}X_{2}X_{3}+b_{24}X_{2}X_{4}+b_{34}X_{3}X_{4}$$

where b_n_/b_nn_ are the constant regression coefficients; Y is the response (i.e., %water content, a_w_, % NaCl, etc.); X_1_, X_2_, X_3_, and X_4_ are the temperature, the oligofructose concentration, the maltodextrin concentration, and the immersion time, respectively [38]. The variables’ levels were chosen based on preliminary experiments. 

In the present study, the aim of the optimization of the OD process was to produce a final product of intermediate moisture that, when submitted to subsequent drying, would reach the desired moisture content more rapidly (decreasing the required drying time). For that reason, appropriate desirability functions were applied in order to estimate the optimized osmotic treatment process parameters, i.e., osmotic solution concentration, including maltodextrin and oligofructose concentration, temperature, and the duration of osmosis, which would give a minimum a_w_ along with maximum color preservation, based on two simultaneous criteria, i.e., maximum lightness retention (L/L_0_) and minimum overall color change (ΔΕ*), setting a minimum acceptable value L/L_0_ of 0.8 and a maximum acceptable value ΔΕ* of 15 (based on a preliminary sensory test, where color change was set as the rejection criterion). To verify the operation performance of the model, an independent validation experiment was carried out and repeated three times.

### 2.6. Air-Drying Process

The osmotically pretreated mushroom stems, derived after OD at optimum conditions, were further dehydrated in an experimental air-dryer (Armfield, Ltd., Hampshire, UK). The samples were placed in the dryers’ perforated tray. The air velocity was kept constant at 1 m/s. The drying process was carried out at three temperature levels of 50, 60, and 70 °C for up to 380 min (50 °C, 60 °C) and 240 min (70 °C) until a constant weight was reached. Since samples dried at increased temperature levels reach equilibrium (constant weight) faster [8], different time intervals during processing at different temperatures were applied. The sample weight was recorded every 10 min for the first hour, every 15 min for the next 2 h, and then every 30 min until a constant weight was reached. The experiments were performed in triplicate.

### 2.7. Air-Drying Kinetics’ Modeling 

For the description of moisture transfer during air-drying, a first-order kinetic model was selected, as follows [8]:

(6) $$-\frac{dX}{dt}=-k\cdot (X-X_{e})$$

where X is the material moisture content (dry basis) during drying (kg water/kg dry solids), X_e_ is the equilibrium moisture content of the dehydrated material (kg water/kg dry solids), k is the drying rate (min^−1^), and t is the time of drying (min). The drying rate is determined by the slope of the falling rate of the drying curve. 

The solution of Equation (6) derived the following expression:

(7) $$X=X_{e}+(X_{i}-X_{e})\cdot e^{-k\cdot t}$$

The effect of process temperature during air-drying was included in the model parameters as follows:

(8) $$k=k_{0}\cdot {\left(\frac{T}{60}\right)}^{k_{1}}$$

where k_0_ is a constant (min^−1^), k_1_ is a dimensionless constant (-), and T is the dry bulb temperature of the air (°C). The temperature was divided by 60 since this was the central temperature value of air-drying used in the experimental design [8].

Non-linear regression analysis was used for parameter estimation using Statistica 12.0 (Stat. Soft Inc., Tulsa, OK, USA).

## 3. Results and Discussion

An analysis of variance (ANOVA) was carried out to determine the statistical significance of the linear, quadratic, and interaction effects of various responses. The results are presented in Table 2. The coefficient of determination (R^2^) measured the goodness of fit of the model. The high values of R^2^ (>0.86) for the %moisture content, a_w_, °Brix, WL, SG, and salt intake (%NaCl) suggested that the corresponding models were reliable enough to anticipate the parameter values. The polynomial equations’ performance for the color parameters, namely, lightness preservation (L/L_0_) and total color change (ΔΕ*), as assessed by the respective R^2^, was lower (>0.78), but they were still deemed capable of predicting the outcomes in a reliable way.

Water loss and solid gain were mostly affected by temperature (X_1_) and oligofructose concentration (X_2_), while temperature (X_1_) and maltodextrin concentration (X_3_) seemed to also affect color parameters (L/L_0_ and ΔΕ*) and salt intake (%NaCl). Shinde and Ramaswamy (2021) [39] also reported that temperature is a significant (*p* < 0.05) factor for the mass transfer parameters WL and SG. Solute concentration (X_2_ and X_22_ for oligofructose and X_3_ and X_33_ for maltodextrin concentration) had a significant effect on water activity, solid gain, salt intake (%NaCl), all color parameters, and texture (F_max_/F_max0_). The cumulatively highest amounts of the two sugars (adding up to a total concentration of about 60–70%) subserved water loss, while lower sugar concentrations (30–40–50%) resulted in less moisture reduction. The same results were observed at all three temperatures. In terms of the osmotic agents applied, during osmosis, the kinetic parameters were strongly affected by the kind of carbohydrates used, especially depending on their molecular weight and ionic behavior. The osmotic pressure of solutions mainly depends on the concentration of low-molecular-weight substances. High-molecular-weight substances produce a lower osmotic pressure, resulting in lower mass transfer parameters and less penetration of the high-molecular-weight substances into the material. Therefore, in our case, the two osmotic agents used, being of a relatively low molecular weight, had a significantly reduced water activity, with οligofructose being slightly more effective than maltodextrin [40,41]. The duration of osmosis had a significant effect on moisture content and chroma (C*). Similar results were observed by González-Pérez et al. (2022) [42] and Seth et al. (2021) [43]. They observed that increasing the solution concentration promoted more significant moisture removal and increased the amount of solutes incorporated into the final product. As for texture, we could observe a lower R^2^ value. This could be explained by the variability in the initial mushroom sample.

### 3.1. Effect of Osmotic Dehydration Process Parameters 

Response surface plots are three-dimensional diagrams that display the impact of two independent variables on a response at once, while maintaining the values of all other variables constant. The surface plots for four dependent variables (% moisture content (%MC), water activity (a_w_), total color change (ΔΕ*), and salt intake (% NaCl)) are illustrated below as the effect of any two of the independent variables (30–50 °C osmosis temperature, 40–80 min of immersion time, 20–40% concentration of oligofructose, and 10–30% concentration of maltodextrin), while the other two parameters are maintained at the center point value. The particular variables were indicatively chosen, as they demonstrated the most significant changes during the process for the time frame selected in the RSM methodology, i.e., 40–80 min. Other parameters such as WL, SG and water activity slightly changed after the 40 min of osmosis in most cases, whereas other quality factors, such as sample lightness and hardness, did not exhibit significant changes during the selected time intervals. 

#### 3.1.1. Effect of Temperature and Time of Osmosis on Dependent Variables

**Figure 2:**
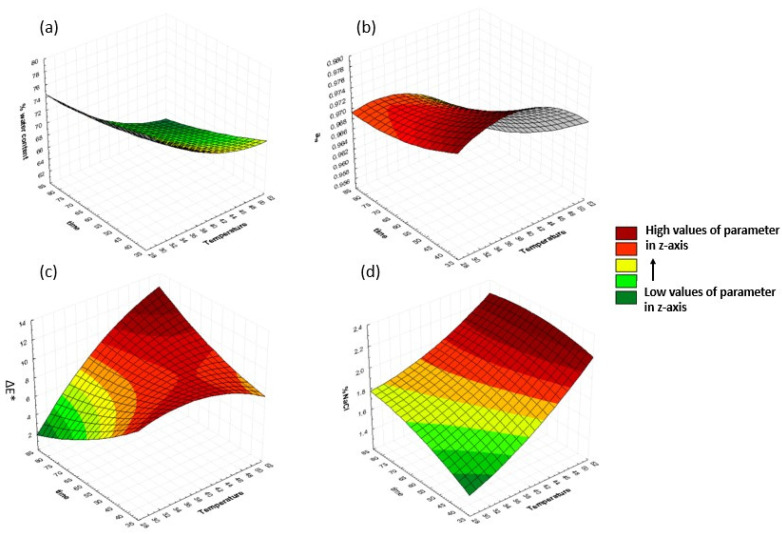
3D response surface graphs for (**a**) %water content, (**b**) a_w_, (**c**) ΔΕ*, and (**d**) %NaCl, as a function of osmosis duration and temperature, at a fixed OD concentration of 30% oligofructose and 20% maltodextrin.

In order to investigate the effect of temperature and process duration on the response variables, the osmotic solution concentration was kept constant at 30% oligofructose and 20% maltodextrin. The results are shown in Figure 2. The moisture content (%MC) decreased with increasing the immersion time and temperature. Lower values were observed at 80 min of osmosis, equal to 63.1%, 66.5%, and 73.3% at 50, 40, and 30 °C, respectively. Water activity decreased with an increase in time and temperature, with lower values being obtained at 80 min of osmosis, equal to 0.9593, 0.9690, and 0.9692 at 50, 40, and 30 °C, respectively. Earlier research [43,44,45,46] also confirmed that a_w_, WL, and moisture content decreased with an increase in the duration and temperature of the process, for citrus, peach, carambola fruit, and banana slides, respectively. Moreover, it could be observed that temperature had a stronger influence on water activity than the duration of the process. On the other hand, the total color change and %NaCl increased with the increase in osmosis time and temperature. Rai et al., in 2022 [46], also reported that ΔΕ* increased with time. The values for the total color change at 30 °C ranged from 4.30 to 8.90, at 40 °C from 7.48 to 9.75, and at 50 °C from 7.68 to 11.82, with lower values corresponding to shorter osmosis times and higher one to higher osmosis times. The highest concentrations of salt were obtained at 50 °C, with values up to 2.05% at the three different osmosis times. Salt concentrations at the other two temperatures (30 and 40 °C) were under 1.86 and 1.90%, respectively. The same results were obtained by Kaur et al. (2022) [45] and Shinde & Ramaswamy (2021) [39]. They reported that temperature had a positive effect, and it was suggested that a rise in temperature would lead to greater water removal and solids’ uptake.

#### 3.1.2. Effect of Dehydration Time and Oligofructose Concentration on Dependent Variables

**Figure 3:**
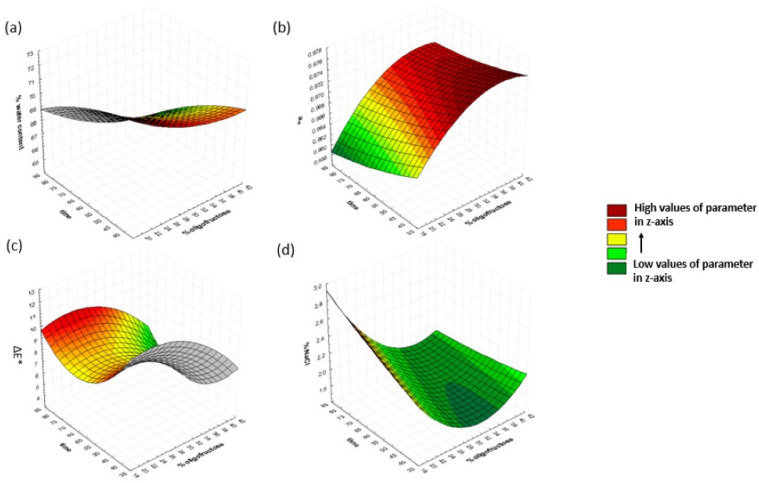
3D response surface graphs for (**a**) %water content, (**b**) a_w_, (**c**) ΔΕ*, and (**d**) %NaCl, as a function of osmosis duration and oligofructose concentration, at a fixed maltodextrin concentration of 20% and a temperature of 40 °C.

**Figure 4:**
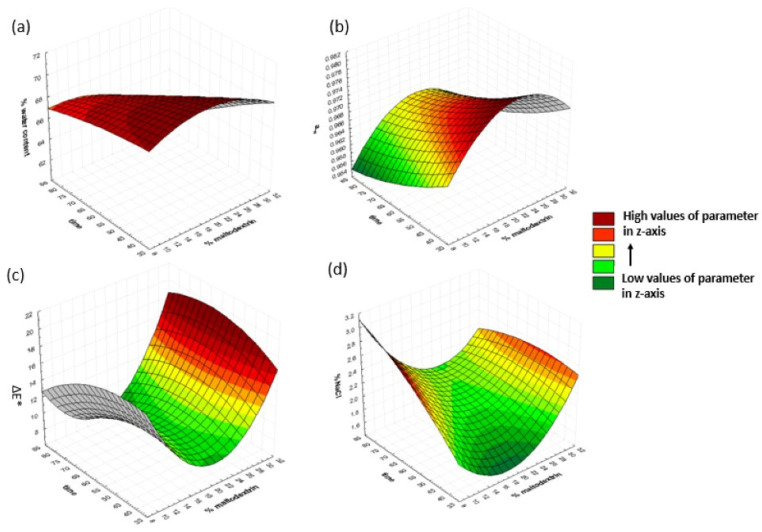
3D response surface graphs for (**a**) %water content, (**b**) a_w_, (**c**) ΔΕ*, and (**d**) %NaCl, as a function of osmosis duration and maltodextrin concentration, at a fixed oligofructose concentration of 30% and a temperature of 40 °C.

To investigate the effect of time and oligofructose concentration on the response variables, osmosis temperature and maltodextrin concentration were kept constant at 40 °C and 20%, respectively. The results are shown in Figure 3. The moisture content decreased with the increase in immersion time and oligofructose concentration. The percentage of moisture at 80 min of osmosis was 68.7%, 66.5%, and 65.7% (on a wet basis) for 20, 30, and 40% oligofructose, respectively. Similar findings were observed by Kaur Dhillon et al. (2022) [44] and Kaur et al. (2022) [45], works in which the authors reported an increase in water removal by increasing the solute concentration. Water activity decreases with the decrease in oligofructose concentration and the increase in immersion time. However, the influence of immersion time was found to be insignificant, based on Figure 4 and an analysis of variance (ANOVA, a = 0.05) applied for the effect of different osmosis times on water activity, possibly due to the close intervals which had been chosen (40, 60, 80 min) for investigation in the RSM scheme. %NaCl decreased with an increase in oligofructose concentration, while there were no significant changes with time. This might have been attributed to the higher solute concentration that formed a coating along the surface of the food sample and, hence, limited the mass transfer phenomena. The same results were observed by Giraldo et al. (2003) [47] on mango due to the high viscosity of the osmotic solution. Rai et al. (2022) [46] also reported a 3% drop in the weight reduction of bananas at the highest concentration studied (65°Brix). Higher values of total color change were observed at lower concentrations of oligofructose. Similar outcomes were reported on bananas [46]. The values ranged from 5.9 to 11.8 at 20% oligofructose, 7.5 to 9.8 at 30%, and 5.0 to 6.76 at 40%. 

#### 3.1.3. Effect of Dehydration Time and Maltodextrin Concentration on Dependent Variables

To investigate the effect of time and maltodextrin concentration on the response variables, osmosis temperature and oligofructose concentration were kept constant at 40 °C and 30%, respectively. The results are shown in Figure 4. The moisture content decreased with increasing maltodextrin concentrations and immersion times. The lower values were found to be 67.4%, 66.5%, and 62.9% at 80 min of osmosis and 30, 40, and 50% maltodextrin, respectively. Water activity decreased with an increase in immersion time and maltodextrin concentration. The salt concentration increased with an increasing dehydration time. The lowest values of salt intake were obtained at 40 min of osmosis and were equal to 1.79, 1.85, and 2.19 at 10, 20, and 30% maltodextrin. Earlier research [42] also confirmed that a reduction in a_w_ was related to the osmotic solution’s concentration. Increasing the osmotic solution’s concentration fosters a more significant moisture reduction. The highest values of total color change (15.5) were observed at the highest concentration of maltodextrin, while no significant changes were observed at different osmosis times. Haque et al. (2020) [48] also observed an increase in color difference on banana samples by increasing the solution concentration.

### 3.2. Optimization and Validation of the Process

**Figure 5:**
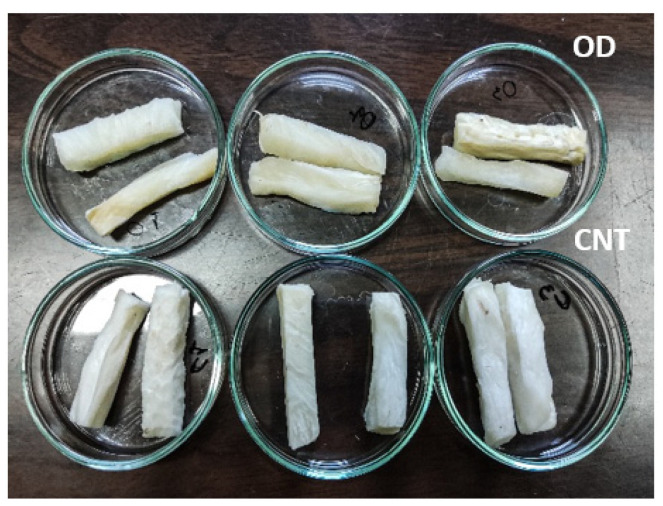
Mushroom by-products after osmosis at the optimum conditions (OD samples) compared to fresh mushroom by-products (CNT samples).

As described in §2.5, proper preset criteria were applied to optimize the OD process. Based on the desirability functions’ approach described by previous studies [38,49], the optimal conditions were found to be 38 °C, 40% oligofructose, 19.3% maltodextrin, and 80 min of immersion time. Three independent experiments (replications) were conducted at the optimum conditions (samples treated, OD are shown in Figure 5 vs. the untreated, CNT) to validate the theoretical values that had been predicted using the second-order polynomial equations developed for all the dependent variables. The theoretical, average of experimental values, and standard deviations are reported in Table 3.

As it can be seen from Table 3, the experimental values were close to the predicted values, with the % error not exceeding 10% in most dependent variables. Hence, the effectiveness of the model in predicting the osmosis parameters could be confirmed to a significant extent. The only exception was texture (hardness change), as described by the relative change in hardness (F_max_/F_max0_), where the absolute error was close to 15%, possibly due to the large variability in the raw material observed. However, it seems that the hardness of the processed mushroom stems was reduced compared to the untreated samples. Similar results had been previously observed for bananas [46].

### 3.3. Hot-Air-Drying Kinetics

#### Effect of Air-Drying Temperature on Drying Kinetics of Optimal Osmodehydrated Mushroom Stems

**Figure 6:**
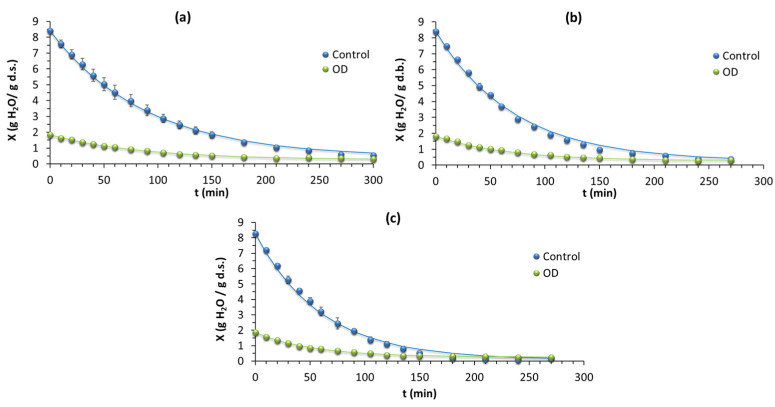
Drying kinetics of fresh and osmotically dehydrated mushroom samples at 50 °C (**a**), 60 °C (**b**), and 70 °C (**c**), where X is the moisture content, in g H_2_O/g d.s. The lines represent the predicted moisture content values for each sample, and the bullets represent the corresponding average experimental values.

The effect of the drying temperature (50, 60, and 70 °C) and duration on the moisture content (on a d.b.) of fresh and osmotically dehydrated mushroom stems at the optimal conditions is presented in Figure 6. The proposed model successfully predicted the air-drying kinetics of osmodehydrated mushroom stems, based on the values of R^2^ (Table 4). As expected, the drying rates were highest at the beginning of drying, as the moisture content had the highest values. The physicochemical changes taking place in the mushroom stems during osmotic dehydration caused differences in the drying rate of the air-drying process. The results showed that increasing the drying temperature shortened the drying time for both the non-pretreated and the pretreated mushroom stems (Table 4 and Figure 6). The values of the rate constants of the pretreated mushroom stems were found to be greater than those of the non-pretreated stems during drying at all the drying temperatures investigated. In addition, the increase in drying temperature for both the pretreated and the untreated mushroom stems caused an increase in the rate constants. This could be attributed to an increase in both the vapor pressure and the diffusion of water with temperature. As it can be seen from Figure 6, a significant reduction in the drying time of the osmodried mushrooms stems could be observed compared to the fresh mushroom stems. Such a reduction in drying time could lead to less energy consumption. Similar results have been reported for banana slices [46] and pumpkins [8]. For fresh mushroom stems, the drying time to achieve the experimentally determined equilibrium moisture content (X_e_), which depends on the drying temperature, at 50 °C (X_e_ = 0.42 g/g d.m.), 60 °C (X_e_ = 0.25 g/g d.m.), and 70 °C (X_e_ = 0.10 g/g d.m.) was 4.5, 4, and 3.5 h, respectively, while, for the osmotically dehydrated mushroom stems, the drying time to achieve an equilibrium moisture content at 50 °C (X_e_ = 0.277 g/g d.m.), 60 °C (X_e_ = 0.279 g/g d.m.), and 70 °C (X_e_ = 0.263 g/g d.m.) was 3.7, 3, and 2.3 h, respectively. 

Moreover, it should be pointed out that, at some point during the drying process, the drying rate constants reached a plateau (X_e_ was achieved). To explain this finding, several factors were involved, such as the presence of a dry layer on the surface of the mushrooms, which hindered the diffusion of moisture from the interior of the mushrooms, as well as the presence of oligosaccharides and salt in this layer. Therefore, it is important to consider both the initial and final moisture content, as well as other parameters such as the surface heat and the mass transfer coefficients, to accurately determine the effect of temperature and osmosis on drying rate constants. Furthermore, the specific type of osmotic solution used may affect the drying rate constants differently. For example, a high concentration of salt may lead to an increase in the drying rate constants, while a high concentration of sugar may slow down the drying rate. In summary, the effect of hot-air temperatures on the drying rate constants in both raw and osmodehydrated mushrooms stems will depend on the initial moisture content, the degree of osmotic pretreatment, and the type and concentration of the osmotic solution previously used.

## 4. Conclusions

In this study, the combined effect of osmotic dehydration at optimal conditions and a hot-air-drying process was investigated in order to design and implement a sustainable application of mushroom by-products. The results showed that the main factor that affected both the mass transfer and the quality characteristics of the mushroom stems during osmotic dehydration was the process temperature. Since the main target of the osmotic pretreatment was water reduction rather than solid uptake, osmotic agents of a low molecular weight were selected, including oligofructose, a well-known prebiotic fiber with significant health benefits. The solute mixture also contained maltodextrin of a low DE, which has been shown to have a positive impact on the quality of finished dried products [22,23]. Studying the first step of our composite procedure (OD followed by air-drying) and aiming to create an osmotically dehydrated product of a minimum a_w_ and with maximum color retention, the optimal conditions for mushroom osmosis were found to be 38 °C, 40% oligofructose, 19.3% maltodextrin, and an 80 min osmotic dehydration duration, based on the RSM results. The efficiency and prediction accuracy of the second-order polynomial equations were confirmed by an independent validation experiment, showing the good performance of the predictive models within the range of the experimental conditions investigated. Osmosis at the optimal conditions was used as a pretreatment, prior to hot-air-drying, to create a dried mushroom product with a reduced drying time along with acceptable sensory characteristics (based on a preliminary sensory assessment of our group), designed for a dried easy-to-carry-and-consume snack. The results showed that osmotic dehydration combined with hot-air-drying led to a modification of the sample matrix properties with a subsequent shelf-life extension. Moreover, the time required to dry the osmotically dehydrated mushroom samples was significantly shorter compared to the time required for the untreated counterparts. Overall, using OD prior to conventional drying led to a modification of the properties of the tissue, an extension of the shelf-life, and less drying time and energy requirements, and, therefore, this combined procedure could provide a basis for the sustainable utilization of mushroom by-products.

## Figures and Tables

**Table 1:** Experimental data for four-variable three-level response surface analysis.

Process Factors	Temperature (°C)	Oligofructose Concentration (%)	Maltodextrin Concentration (%)	Duration of Osmosis (min)			
High	50	30	40	80	+1	+1	+1
Center	40	20	30	60	0	0	0
Low	30	10	20	40	−1	−1	−1
Standard order	X_1_	X_2_	X_3_	X_4_			

**Table 2:** Constant regression coefficients (b_i_, b_ij_, and b_ii_) of Equation (5) (RSM) and statistical analysis using Analysis of Variance (ANOVA).

Constant Regression Coefficients	% MC	a_w_	°Brix	WL	SG	L/L_0_	ΔΕ*	NaCl (%)	C*	F_max_/F_max0_
Intercept	115.0311 *	0.948488 *	22.92045 *	6.423953	12.17969 *	−0.121860	61.42830 *	−1.53541	11.96114 *	−5.30788
X_1_	−1.0140	0.000287	−0.22931	−0.232524 *	−0.31859 *	0.033076 *	−1.48643 *	0.18600 *	0.19815	0.08266
X_11_	0.0157 *	0.000002	−0.00011	0.003764 *	0.00283 *	−0.000221	0.01128	−0.00249 *	−0.00263	−0.00141
X_2_	−0.6042	0.002043	0.69079 *	−0.030746	−0.17630 *	0.002328	−0.45083	−0.00745	−0.2185 *	0.12323
X_22_	0.0117	−0.000026	0.00110	0.000497	0.00201 *	0.000010	0.00071	0.00001	0.00190	−0.00329 *
X_3_	−0.3165	0.001547	0.30553	0.090251	−0.27120 *	0.029644 *	−1.32513 *	−0.03631	−0.7937 *	0.32601 *
X_33_	−0.0053	−0.000051 *	0.01082 *	0.001125	0.00095	−0.000560 *	0.02026 *	0.00209 *	0.0129 *	−0.00422 *
X_4_	−0.0067	−0.000372	−0.01658	−0.003637	0.00025	0.002970	−0.06856	0.01040	0.02313	−0.01646
X_44_	0.0006	0.000002	0.00003	0.000046	0.00001	−0.000010	−0.00003	0.00002	−0.00061	−0.00002
X_12_	−0.0138 *	−0.000031 *	0.00458	−0.000101	0.00021	−0.000248	0.00956	0.00129	0.00191	0.00183
X_13_	0.0066	0.000007	0.00333	−0.003618 *	0.00447 *	−0.000392 *	0.01237 *	−0.00085	−0.00567	−0.00211
X_23_	0.0053	0.000008	0.00250	−0.000200	0.00259 *	0.000350 *	−0.00074	−0.00147	0.00830	−0.00125
X_14_	−0.0030	−0.000001	0.00071	0.000198	0.00001	−0.000028	0.00169	0.00017	0.00162	0.00056
X_24_	−0.0009	0.000000	−0.00075	−0.000200	0.00013	0.000001	−0.00034	−0.00035	−0.00270	0.00029
X_34_	−0.0002	0.000002	−0.00000	0.000270	−0.00001	−0.000040	0.00005	−0.00012	0.00337	−0.00055
R^2^	0.922661	0.902627	0.995198	0.862179	0.871397	0.780621	0.797130	0.862261	0.8178	0.705193
R^2adj^	0.797926	0.653159	0.986981	0.620296	0.669390	0.516289	0.620557	0.614023	0.6078	0.490638
R^2predict^	0.851304	0.763268	0.990420	0.717199	0.742004	0.603307	0.730032	0.717677	0.7037	0.530847

X_1_, temperature (°C); X_2_, % oligofructose concentration; X_3_, % maltodextrin concentration; X_4_, duration of osmosis (min). * *p*-value < 0.05; values assigned an asterisk are statistically significant coefficients at a level of 95%.

**Table 3:** Predicted and experimental values for the responses at the optimum conditions.

	Predicted Values	Experimental Values	Error (%)
a_w_	0.9768	0.9705 ± 0.01	−0.64
WL	2.15	2.38 ± 0.16	9.66
SG	1.57	1.60 ± 0.22	1.88
ΔE*	8.18	8.44 ± 0.97	3.05
L/L_0_	0.95	0.92 ± 0.02	−3.35
%NaCl	2.10	2.01 ± 0.38	−4.57
°Brix	61.30	61.50 ± 0.87	0.29
%MC	63.57	64.55 ± 1.37	1.51
C*	4.09	4.71 ± 0.88	13.16
F_max_/F_max0_	0.67	0.58 ± 0.28	−15.07

**Table 4:** Results of parameter estimation for the mathematical model of air-drying kinetics of fresh and OD mushroom stems.

Fresh Mushroom Stems		R^2^
K_0_	0.0140 ± 0.0001	0.997
K_1_	1.1552 ± 0.0673	
Osmodehydrated Mushroom Stems		R^2^
K_0_	0.0152 ± 0.0001	0.996
K_1_	1.3228 ± 0.0709	

## Data Availability

The original contributions presented in the study are included in the article, further inquiries can be directed to the corresponding author.
